# In vitro diagnostics in hymenoptera venom allergy: Current concepts and perspectives 

**DOI:** 10.5414/ALX02639E

**Published:** 2026-08-25

**Authors:** Thilo Jakob, Timo Buhl, Jörg Fischer

**Affiliations:** 1 Department of Dermatology and Allergy, University Medical Center Gießen, Justus Liebig Universität Gießen, Gießen,; 2 Department of Dermatology, Venereology and Allergology, University Medical Center Göttingen, Göttingen, and; 3 Department of Dermatology and Allergology, Augsburg University Hospital, Augsburg, Germany

**Keywords:** insect sting anaphylaxis, immunoglobulin E, allergens, component resolved diagnostics, ratio analysis, tryptase, cKit

## Abstract

Background: Hymenoptera venom allergy (HVA) is a leading cause of anaphylaxis in adults and requires precise diagnostic work-up to guide venom immunotherapy (VIT). However, the high prevalence of asymptomatic sensitization and frequent double sensitization complicate the identification of clinically relevant allergens. Materials and methods: This narrative review summarizes current concepts and recent advances in in vitro diagnostics of HVA, including conventional IgE testing, component-resolved diagnostics (CRD), IgE ratio analysis, and cellular assays and biomarkers for risk stratification. Emphasis is placed on their diagnostic performance, limitations, and clinical applicability. Results: Measurement of venom-specific IgE remains the cornerstone of HVA diagnosis, offering high sensitivity for clinically relevant HVA. CRD using recombinant, CCD-free allergens improves discrimination between primary sensitization and cross-reactivity, particularly in patients with double sensitization to honeybee and yellow jacket venoms. However, incomplete allergen panels, especially in honeybee venom allergy, limit sensitivity. IgE ratio analysis has emerged as a complementary tool to identify the most likely culprit venom, although it may be influenced by recent sting exposure. Cellular assays such as the basophil activation test provide functional information and may support diagnosis in complex cases but are restricted to specialized centers. In addition, biomarkers such as basal serum tryptase and KIT p.D816V mutation are important for risk stratification. Conclusion: Modern in vitro diagnostics substantially enhance the precision of HVA diagnosis but cannot replace clinical history. An integrated approach combining serology, molecular diagnostics, ratio analysis and, where appropriate, functional assays, is essential for accurate identification of the relevant venom and optimal patient management.

## Introduction 

Hymenoptera venom allergy (HVA) is one of the most common causes of anaphylaxis in adults and is frequently associated with severe anaphylaxis [1]. It results in significant morbidity and impairment in quality of life [2]. A prevalence of up to 3.5% is reported in Germany [3] and Europe [4]. Causal treatment in the form of venom immunotherapy (VIT) is highly effective and well tolerated [5]. 

In Germany and Northern Europe, the main elicitors of HVA are yellow jackets *(Vespula)* and honeybees *(Apis).* Bumblebees *(Bombus)* and hornets *(Vespa)* are less frequently involved, and allergic reactions are often attributed to cross-reactivity to the primary sensitization to honeybee venom (HBV) and yellow jacket venom (YJV). In the Americas and Mediterranean regions paper wasps (*Polistes*), white-faced hornets (*Dolichovespula*), and stinging ants (*Formicidae*) are additional relevant elicitors of sting anaphylaxis but these are currently of little or no significance in Germany and Northern Europe. 

According to the current guideline for management of bee and wasp venom allergy, only patients with a clinical history of anaphylactic sting reactions should undergo diagnostic testing for hymenoptera venom allergy, and only those with evidence of IgE-mediated sensitization to hymenoptera venom should be offered VIT [5]. 

The prevalence of IgE-mediated sensitization to HBV and/or YJV in the general population is high, ranging from 42 – 55% depending on the cutoff used for sIgE detection [3] and even higher (49 – 73%) in individuals with an increased insect sting exposure due to outdoor activities such as fishing and hunting [6]. The sIgE response to insect venoms (and other venoms) has been reported to be part of a beneficial physiological response that allows for an improved inactivation of venom components by enhanced release of mast cell proteases [7, 8]. Thus, sIgE to a given insect venom primarily reflects prior sting exposure and should not be interpreted as evidence of venom allergy in the absence of a compatible clinical history. 

**Figure 1.**
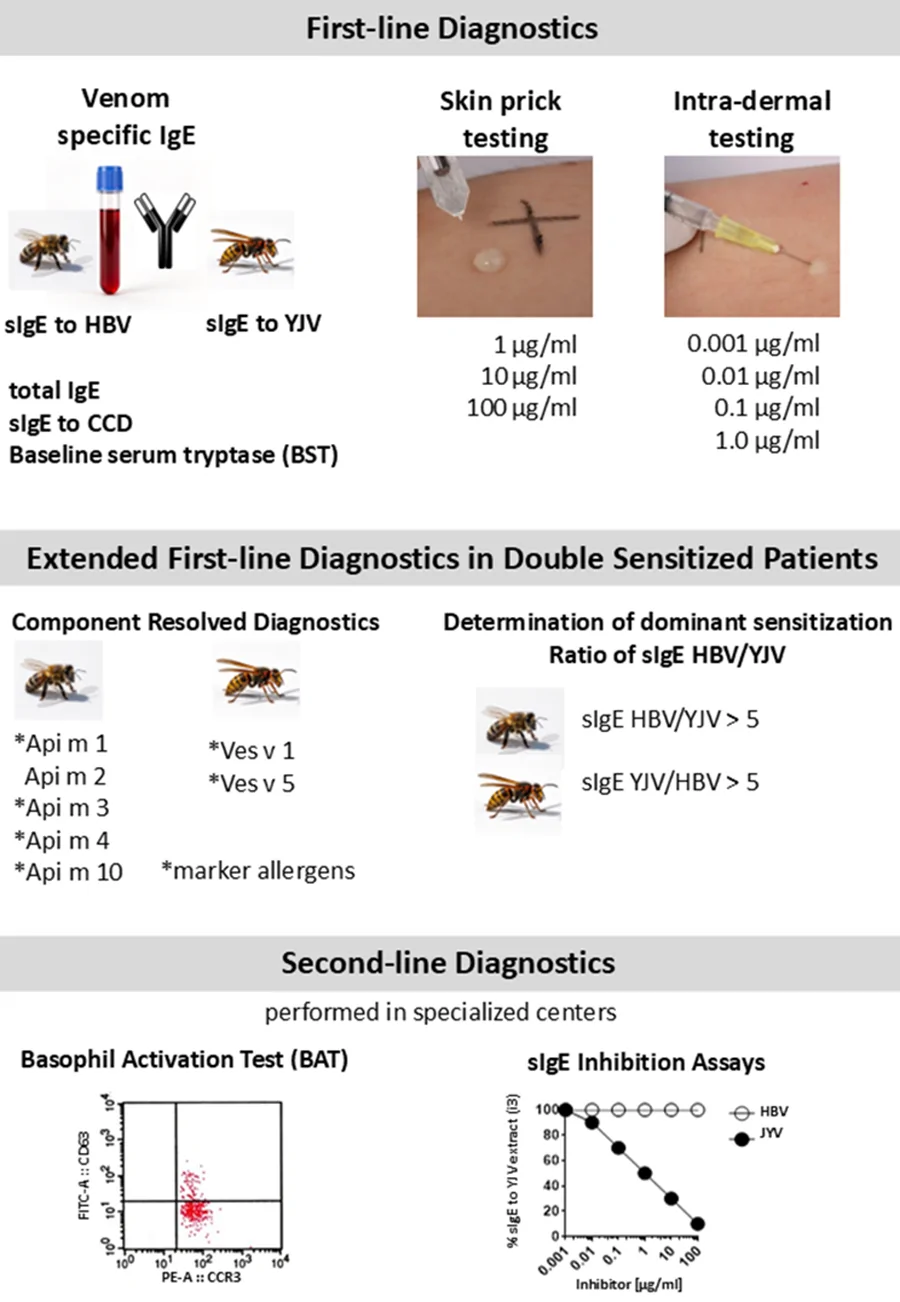
Current tools available for diagnostics in hymenoptera venom allergy.

Several factors complicate the diagnostic process in HVA. The high rate of asymptomatic hymenoptera venom sensitization in the general population, failure to identify the culprit insect, decline or loss of sensitization over time, and conditions mimicking anaphylaxis can all lead to an incorrect diagnosis. Up to 50% of those allergic to hymenoptera venom are double sensitized to HBV and YJV, but usually only one of these sensitizations is clinically relevant [9]. Often the insect responsible for the systemic reaction goes unidentified. When the insect was identified, it should be noted that the ability of the general population to correctly identify hymenoptera is limited [10]. In order to minimize the risks of not detecting clinically relevant sensitizations, reliable diagnostic tests that accurately identify hymenoptera venom sensitizations are essential. The present review summarizes the diagnostic tools in hymenoptera venom allergy (Figure 1) and discusses current concepts and recent advances in the field. 

## Detection of IgE-mediated sensitization to hymenoptera venom 

Detection of IgE-mediated sensitization to hymenoptera venom can be obtained by skin testing and/or measurement of sIgE in the peripheral blood. 

### Skin testing 

Skin prick tests (SPT) are simple, rapid, and inexpensive, commercially available venom solutions (up to 100 µg/mL) that show limited sensitivity, particularly for HBV, and reduced specificity at higher concentrations [11]. Intradermal (i.c.) testing with concentrations up to 1 µg/mL provides higher sensitivity and specificity but requires preparation from therapeutic venom extracts, as no commercial i.c. solutions exist. The safety and efficacy of simultaneous intra-dermal testing in 478 hymenoptera venom allergic patients with 0.02 mL of 0.001, 0.01, 0.1, and 1.0 μg/mL of HBV and YJV were assessed. A systemic reaction incidence of 0.6% was reported [12], but no severe reactions occurred. A more recent study of 300 patients with suspected HVA in which skin testing consisted of simultaneous intra-dermal tests with 0.02 mL of 1.0 µg/mL of 5 different commercially available venom preparations reported no immediate and only 1 delayed adverse reaction [13]. The current guideline on the diagnosis and management of HVA [5] recommends the use of skin testing in HVA, however it places it secondary to sIgE testing as indicated by the following recommendation: 

*“A skin test may be omitted if there is a particular risk associated with the skin test, if its performance would severely affect the patient, and/or if a clear result has already been obtained from in-vitro tests. Skin testing should be performed if the sIgE diagnosis is negative or if there is a discrepancy between the history and in-vitro findings”.*

### Detection of hymenoptera venom–specific IgE 

The assessment of venom-specific IgE represents the most commonly applied method to confirm IgE-mediated sensitization in patients with systemic reactions to insect stings. A variety of assay formats are available, including liquid- and solid-phase systems as well as singleplex and multiplex approaches. Reported sensitivities for honeybee venom (HBV)-specific IgE are high, ranging from 98 – 100% in HBV-allergic individuals [14, 15], whereas sensitivities for yellow jacket venom (YJV) are somewhat lower (83 – 93%) [14, 15, 16]. It should be noted that measures such as diagnostic specificity or positive predictive value are of limited relevance in this context, as sIgE testing identifies sensitization but does not establish clinical relevance. 

Venom-specific IgE can, in principle, be measured at any time following a sting event. However, taking advantage of the immunological booster effect, testing is optimally performed 3 – 6 weeks after the sting reaction [5, 17, 18]. Failure to detect sIgE despite a convincing clinical history may be explained by long intervals between the sting event and diagnostic evaluation. Serum sIgE levels have been shown to decline within 1 – 4 years after anaphylaxis and may eventually fall below detection thresholds. Earlier assumptions suggesting consumption of sIgE during anaphylaxis have not been substantiated. 

The conventional cut-off for sIgE positivity is 0.35 kU_A_/L, although modern assay systems allow reliable detection down to 0.10 kU_A_/L [19]. Given the relationship between specific and total IgE, values between 0.10 and 0.35 kU_A_/L may still be clinically meaningful, particularly in patients with low total IgE levels [9]. Importantly, results obtained from different commercial platforms are not directly comparable due to substantial inter-assay variability, partly related to differences in the heterologous calibration used [20, 21]. Therefore, current guidelines recommend clearly specifying the assay system used in clinical reporting [5]. 

## Molecular allergy diagnostics in insect venom allergy 

Approximately 45 – 50% of patients with hymenoptera venom allergy exhibit double sensitization to honeybee venom (HBV) and yellow jacket venom (YJV) when assessed by venom-specific IgE. This finding complicates the identification of the clinically relevant venom, particularly if the culprit insect is unknown [22]. Double sensitization may arise from (i) true sensitization to both venoms following independent stings, (ii) IgE cross-reactivity to homologous venom proteins, or (iii) IgE directed against cross-reactive carbohydrate determinants (CCD) shared by multiple allergens [23]. Importantly, the presence of CCD-specific IgE does not exclude clinically relevant sensitization to protein epitopes [22]. 

### Discrimination between HBV and YJV by component-resolved diagnostics 

The introduction of recombinant allergen technology has enabled the production of CCD-free molecules, thereby substantially improving diagnostic specificity [24]. Consequently, component-resolved diagnostics (CRD) has become an essential tool in the work-up of hymenoptera venom allergy. Currently, the most relevant allergens include rVes v 1 and rVes v 5 for YJV, and rApi m 1, rApi m 2, rApi m 3, Api m 4 (research use), rApi m 5, and rApi m 10 for HBV. The analysis of individual allergen components has facilitated the identification of species-specific marker allergens, allowing a more reliable distinction between primary sensitization and cross-reactivity. Ves v 1 and Ves v 5 are established markers for YJV sensitization, whereas Api m 1, Api m 3, Api m 4, and Api m 10 indicate primary HBV sensitization. In contrast, several allergens display considerable cross-reactivity due to sequence homology, including hyaluronidases (Api m 2, Ves v 2), dipeptidyl peptidases (Api m 5, Ves v 3), and vitellogenins (Api m 12, Ves v 6) [25, 26, 27, 28]. Notably, cross-reactivity of hyaluronidases appears to be largely mediated by CCD-specific IgE, as demonstrated for Ves v 2 variants with and without glycosylation [28]. In contrast, IgE reactivity to CCD-free Api m 2 is relatively frequent (43 – 52%) in HBV-allergic patients [29]. Structural analyses further indicate that, despite sequence similarity, Api m 2 and Ves v 2 differ substantially in surface epitope architecture [30]. Thus, sIgE to Api m 2 may, with a grain of salt, support the diagnosis of primary HBV sensitization. 

### Diagnostic performance of individual allergen components 

Initial studies using early liquid-phase assays reported sensitization rates of 97% to Api m 1 in HBV-allergic patients and 87% to Ves v 5 in YJV-allergic patients [15]. Subsequent investigations with current assay systems demonstrated somewhat lower sensitization rates. For YJV, reported frequencies range from 84 – 90% for rVes v 5 and 33 – 54% for rVes v 1. Combined testing for both components increases diagnostic sensitivity to 92 – 98% [16, 31, 32]. The additional measurement of Ves v 2 and Ves v 3 has been proposed to further enhance sensitivity [33], although this could not be confirmed in a follow-up study [34]. 

In HBV allergy, sensitization to Api m 1 is less frequent than initially reported, with rates between 58% and 80% in studies using current platforms [18, 31, 35, 36]. Thus, the absence of Api m 1-specific IgE does not exclude HBV sensitization. Variability in reported sensitization rates has been attributed to regional differences, patient selection, and assay-dependent factors [18, 31]. Comparative analyses indicate that assay performance differs between platforms. A direct comparison of sIgE levels to Api m 1 measured on the Immulite fluid phase test system and the ImmunoCAP solid phase test system suggested a higher sensitivity for the Immulite system [37, 38]. It was speculated that IgE binding capacity of the recombinant Api m 1 used in the ImmunoCAP system may be diminished due to altered protein folding [37, 38]. However, this assumption was not supported by a direct comparison of IgE binding to purified natural Api m 1, which was found to be identical in CCD negative sera [39]. A more likely cause is an apparent difference in the heterologous calibration system between the two assays, as suggested by one study [38, 40]. Indeed, experimental data suggest that Immulite may overestimate sIgE levels by approximately three- to five-fold [41, 42], potentially explaining higher rates of positive results. 

Beyond Api m 1, additional major allergens contribute to the sensitization profile in HBV allergy. Testing a broader panel including Api m 1–5 and Api m 10 enabled the detection of up to 94% of HBV-allergic patients and highlighted the heterogeneity of sensitization patterns [43]. Notably, Api m 3 and Api m 10 improved the detection of primary HBV sensitization in approximately two thirds of Api m 1-negative cases [44], thereby reducing the diagnostic gap. Despite the availability of multiple recombinant allergens for routine diagnostics – including rApi m 1, rApi m 2, rApi m 3, Api m 4 (available for research use), rApi m 5, and rApi m 10 - the current component panel does not yet allow comprehensive detection of all HBV sensitizations. In a follow-up study, the combined use of these allergens resulted in an overall sensitization rate of ~ 79% among HBV-allergic patients [45], indicating that a relevant proportion of patients remain undetected by current molecular diagnostics. This discrepancy compared to earlier reports [43] may be explained by differences in study populations, including variations in clinical phenotype, geographic exposure, or selection criteria. These findings underscore that, although component-resolved diagnostics have significantly improved the precision of hymenoptera venom allergy diagnostics, it still cannot fully replace conventional testing with whole venom extracts. Rather, CRD should be considered a complementary approach, particularly valuable in cases of double sensitization or inconclusive standard diagnostics. 

**Figure 2.**
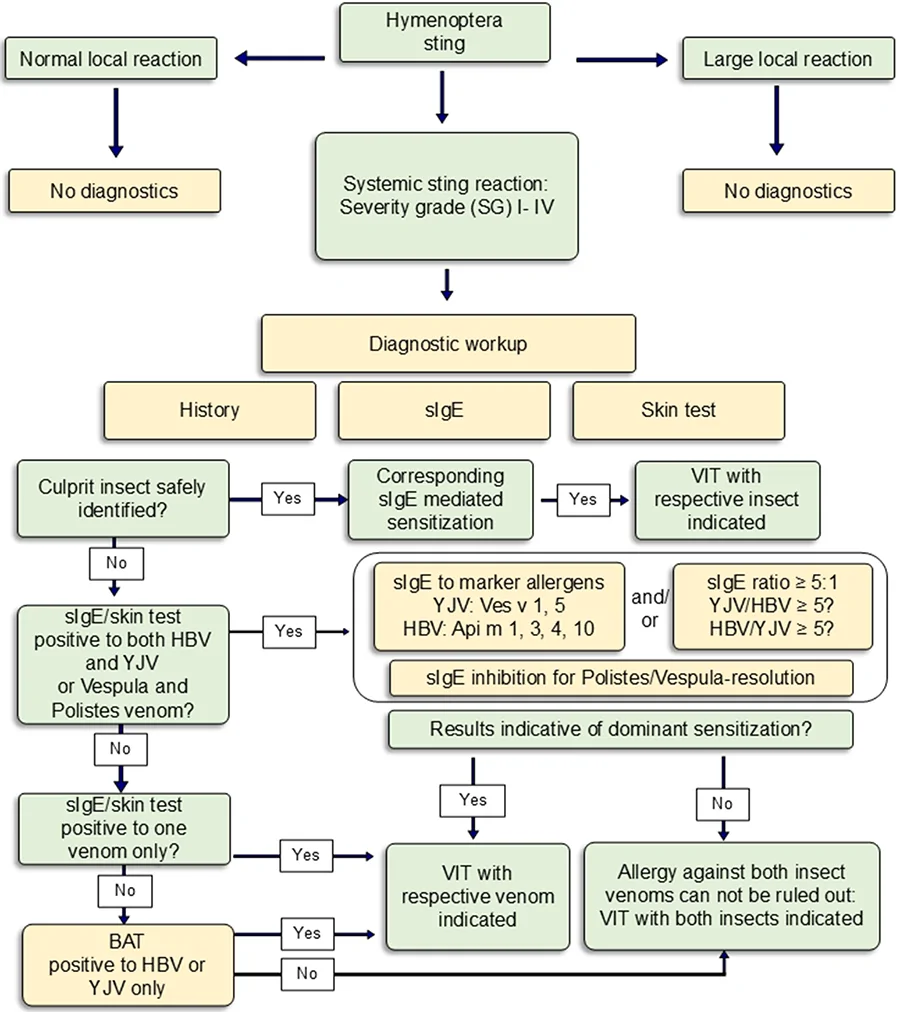
Schematic workflow for the diagnosis of hymenoptera venom allergy (adapted from [69]).

In conclusion, the currently available recombinant hymenoptera venom allergens are useful for the identification of primary sensitizations to YJV and HBV allergens, not confounded by CCD reactivity and have become an integral part of the diagnostic workflow (Figure 2) for the diagnosis of Hymernoptera venom allergy, in particular in patients that display double sensitization where the culprit insect was not unequivocal identified. While Ves v 1 and Ves v 5 negative results exclude YJV sensitization with a high likelihood, negative results to the HBV-specific allergens Api m 1, Api m 2, Api m 3, Api m 4 and Api m 10 do not necessarily exclude HBV sensitization, since a relevant minority (5 – 20%) of HBV allergic patients is not sensitized to any of the available HBV allergens. Here, consideration of low (0.1 – 0.35 kUA/L) IgE levels to HBV marker allergens, comparison of IgE levels to cross-reactive allergens, or the identification of additional HBV marker allergens would be helpful towards optimizing the diagnostic precision. 

### Discrimination between Vespid and Polistes sensitization 

Even more difficult than resolution between HBV and YJV sensitization is the discrimination between sensitization to YJ and Polistes venoms, which is of particular relevance in Mediterranean countries and the Americas. Due to a high degree of IgE cross-reactivity, unequivocal discrimination is rarely achieved. Interestingly, Polistes venom proteins are devoid of CCD reactivity [46], so that cross-reactivity is mostly caused by homologous proteins as described for the hyaluronidases (Ves v 2 and Pol d 2), for the dipeptidyl peptidases (Ves v 3, Pol d 3), the group 5 antigens (Ves v 5, Pol d 5), and to a lesser extent for group 1 antigens (Ves v 1, Pol d 1). Which of these venoms is the most likely primary sensitizer may however be indicated by the relative level of sensitization to them. To this end, the combination of group 1 and group 5 allergens was reported to identify the most probable sensitizing insect in two thirds of the patients studied, while the hyaluronidases (Ves v 2 and Pol d 2) did not provide any additional value [47]. Currently available allergens for routine sIgE diagnostics to YJ and Polistes venom include rVes v 1, rVes v 5, rPol d 1, and rPol d 5. However, no major marker allergens that would support definitive discrimination of sensitizations to venoms of different Vespidae subfamilies have so far been identified [29]. Therefore, sIgE inhibition assay is used as attempt to discriminate between primary Polistes and YJ sensitization. However, they are resource and work intensive, often difficult to interpret, and are only performed in specialized centers [5, 9]. 

## Discrimination between HBV and YJV sensitization by sIgE ratio analysis 

**Figure 3.**
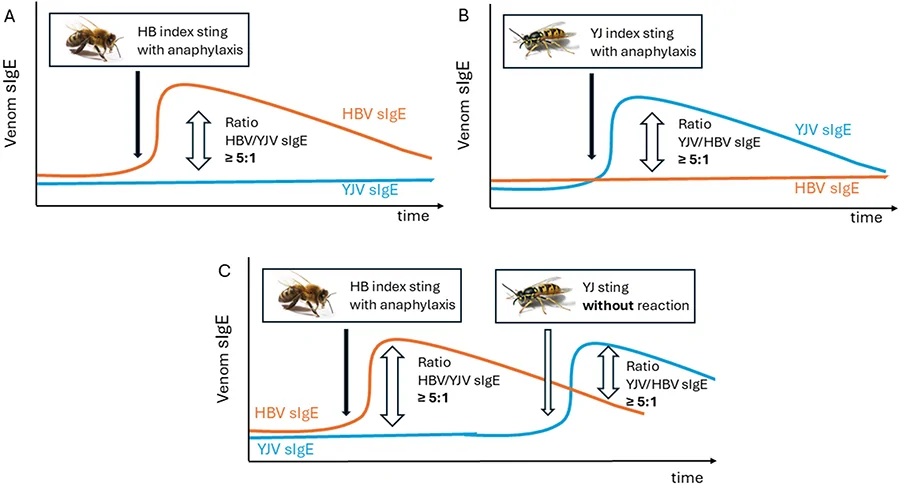
Visualization of sIgE responses in HBV and YJV double-sensitized individuals. Based on the concept that sIgE levels primarily represent sting exposure, index stings are likely to boost the existing sIgE levels leading to higher levels of sIgE to the culprit insect as compared to sIgE to the not relevant insect (A, B). A ratio of > 5 : 1 has been suggested as relevant cut off that the dominant sIgE indicates the relevant venom. In double-sensitized individuals, stings from hymenoptera, to which the patient is not allergic, may equally boost the sIgE level, alter the ratio, and give rise to false-positive ratio for the non-relevant venom (C).

More recently, a different approach has been suggested for the identification of the relevant sensitizing venom in HBV and YJV double-sensitized patients in whom the culprit insect could not be identified. This concept is based on the assumption that sIgE to a given insect venom primarily reflects sting exposure and that the most recent sting induces a booster effect, leading to a more pronounced increase in sIgE to the venom of the responsible insect than to venoms not involved in the index sting. The bee/wasp sIgE ratio described by Tischler et al. operationalizes this concept by dividing the sIgE value of the presumed dominant venom by that of the non-dominant venom [48]. A ratio greater than 5 : 1 is considered supportive of monoallergy and may justify initiating venom immunotherapy directed against the dominant venom alone (Figure 3A, B). Alternative species-specific ratio with vespid/bee ratio > 5.33 and vespid/bee-ratio ≥ 2.68 was suggested by Arzt-Gradwohl et al. [49]. Of note, boostering of venom sIgE following stings from hymenoptera, to which the patient is not allergic, may alter the ratio and give rise to false positive ratio for the non-relevant venom. Therefore, when using the ratio analysis, it is mandatory to specifically ask the patient about additional hymenoptera stings after the index sting that may influence the sIgE ratio (Figure 3C). 

A second essential diagnostic element is the comparison of serological findings with skin test reactivity. Intradermal tests detect sensitization with high sensitivity, and differences in reactivity thresholds between venoms may provide additional orientation. A negative skin test to the non-dominant venom, despite detectable sIgE, strengthens also the assumption of monoallergy. Conversely, titrated intradermal testing with concentrations ranging from 0.001 µg/mL to 1 µg/mL can help identify clinically relevant sensitization when serological results remain ambiguous. In clinical practice, the combination of IgE ratio analysis and skin test comparison often allows reliable attribution of the culprit venom. Dual venom immunotherapy should be reserved for cases in which neither approach, nor the patient’s history, permits a confident assignment [50]. 

## Cellular assays for detection of IgE-mediated sensitization to venom allergens 

Cellular assays represent second-line diagnostic tools in hymenoptera venom allergy and should be reserved for selected cases in which conventional diagnostic approaches do not yield a conclusive result (Figures 1, 2) [51, 52]. The rationale for such functional testing is that conventional assays document IgE sensitization, but do not necessarily establish its functional relevance. By reflecting allergen-induced effector cell activation, cellular assays may help clarify whether a sensitization pattern is clinically meaningful and thus add disease-specific information beyond sensitization testing alone [51]. In a prospective study, BAT showed good diagnostic performance and was explicitly positioned as an add-on test in diagnostically unclear cases [52]. A second clinically important indication is double sensitization to honeybee and vespid venoms. In such patients, BAT may contribute to identifying the clinically more relevant sensitization when extract-based and molecular results remain difficult to interpret. Importantly, double positivity in sensitization testing should not be equated with clinically relevant double allergy. Earlier work showed that BAT, combined with recombinant allergen-based IgE testing and assessment of CCD reactivity, can improve culprit venom attribution beyond extract-specific IgE alone [53]. More recent studies further support its value in double-sensitized patients, including cases in which BAT with venom components supported the choice between single- and dual-venom immunotherapy and helped distinguish true double sensitization from cross-reactivity or otherwise indeterminate findings [54, 55, 56]. Broader implementation is limited by restricted availability, the need for fresh blood samples, inter-laboratory variability, and incomplete standardization. BAT is therefore best regarded as an adjunctive test performed in specialized centers [51, 52]. 

## Risk markers for severe hymenoptera venom anaphylaxis 

IgE-based tests are essential for demonstrating sensitization to hymenoptera venom, but they do not predict the severity of either previous or future sting reactions. Neither the presence nor the magnitude of venom-specific IgE, whether measured against whole venom extracts or individual components, should be interpreted as a surrogate marker of clinical risk. Accordingly, therapeutic decisions are not based on IgE values alone, but on the combination of a clinically relevant systemic sting reaction and evidence of IgE-mediated sensitization. Risk stratification therefore requires additional laboratory markers beyond conventional sensitization testing [57, 58] (Table 1). 

Among these, basal serum tryptase (BST) is one of the most established laboratory parameters associated with severe hymenoptera venom-induced anaphylaxis (Table 1). Its principal value lies less in confirming venom allergy itself than in identifying patients in whom an increased mast cell burden or an underlying mast cell disorder should be considered. Elevated BST has long been associated with more severe systemic sting reactions and should prompt further evaluation, particularly in patients with cardiovascular sting reactions in the absence of skin symptoms or otherwise disproportionate reaction severity [58, 59, 60]. 

This is particularly relevant in clonal mast cell disorders. Mastocytosis and KIT p.D816V-associated clonal mast cell disease are strongly associated with severe hymenoptera venom anaphylaxis and are of major practical importance because affected patients may present with strikingly severe reactions despite only modest or even unremarkable findings in routine allergy tests. Seminal work demonstrated a high prevalence of clonal mast cell disease among patients with hymenoptera sting anaphylaxis and elevated BST, thereby establishing the clinical relevance of bone marrow evaluation in selected cases [59]. More recent studies have shown that routine screening for KIT p.D816V can identify clonal disease that would be missed if BST values were used as the sole screening tool, and that KIT positivity is an important independent marker of severe reactions [61]. In addition, large contemporary cohorts confirm a substantial burden of clonal mast cell disorders among patients requiring venom immunotherapy, underscoring that mast cell pathology is not a rare comorbidity but an important determinant of risk in this population [57, 62]. Importantly, a normal BST level does not reliably rule out clinically relevant clonal mast cell disease in patients with severe sting anaphylaxis [59, 61]. 

Hereditary α-tryptasemia has emerged as a further modifier of anaphylaxis risk. This inherited increase in alpha-tryptase copy number may contribute to persistently elevated BST levels and appears to increase the likelihood of severe venom-triggered anaphylaxis, both independently and in conjunction with mastocytosis [63, 64]. However, its precise clinical weight still requires careful interpretation. Current evidence supports considering hereditary α-tryptasemia particularly in patients with severe sting reactions and elevated BST in whom overt mastocytosis is not found, or in those in whom reaction severity appears disproportionate to the routine allergy work-up [60, 65, 66, 67]. 

In practical terms, BST, mastocytosis, KIT pD816V-associated clonal disease, and hereditary alpha-tryptasemia do not replace allergy testing and do not establish venom allergy but refine risk stratification beyond sensitization testing alone. Their main clinical value is to identify patients who require further evaluation, closer counseling, and particularly careful long-term management, including implications for venom immunotherapy and follow-up [58, 62, 68]. 

Thus, in hymenoptera venom allergy, risk stratification extends beyond the demonstration of IgE-mediated sensitization and should incorporate mast cell-related biomarkers and disorders, particularly in patients with a history of severe systemic sting reactions. 

## Authors’ contributions 

TJ: First and corresponding author, manuscript concept, main editing, main revision, management of references. JF: Contributing author of the section on “Discrimination between HBV and YJV sensitization by sIgE ratio analysis”. TB: Contributing author of the section on “Cellular assays for detection of IgE-mediated sensitization to venom allergens” and “Risk markers for severe Hymenoptera venom anaphylaxis”. All authors have contributed to the development of the figures and tables, and approved the final version of the manuscript. 

## Funding 

None. 

## Conflict of interest 

T.J. reports lecture fees from Alk Abelló, Allergy Therapeutics, Bencard, HAL, Leo, Novartis, and ThermoFisher Scientific, honoraria for advisory boards from Alk Abelló, Bencard, Galderma, Leo, and Novartis as well as research grants from Allergopharma, Germany; ALK-Abello, Denmark; Novartis, Germany; and Thermo Fisher Scientific/Phadia, Germany. TJ is board member of the German Society of Allergy and Clinicial Immunology (DGAKI) and the German Society of Dermatology (DDG) and is Editor-in-Chief of the AllergoJournal and AllergoJournal International. 

T.B. has been a speaker/investigator/advisor for AbbVie, ALK Abelló, Almirall, AstraZeneca, Bencard, Eli Lilly, Incyte, Galderma, HAL, Janssen, Kiniksa, LEO Pharma, Novartis, R-Biopharm, Sanofi, and Thermo Fisher. 

J.F. reports lecture fees from ALK-Abelló, Bencard, Bristol Myers Squibb, Novartis, Janssen-Cilag, Sanofi-Aventis, AeDA (Association of German Allergologists), and GEKA (Society for Experimental and Clinical Respiratory Research), consulting fees from Bencard and Sanofi-Aventis, and travel grants from Pierre Fabre Oncology outside the scope of the submitted paper. 

**Table 1.** Mast cell-related risk factors in severe hymenoptera venom anaphylaxis.

**Factor**	**Typical clinical context**	**Practical implication**
Basal serum tryptase elevation	Severe systemic sting reaction, especially with cardiovascular involvement	Suggests increased mast cell burden or an underlying mast cell disorder and should prompt further evaluation
Mastocytosis/clonal mast cell disease	Severe reactions that appear disproportionate to routine allergy test findings	Identifies patients at increased risk who require careful long-term management
KIT D816V positivity	Severe hymenoptera venom allergy, including cases with normal or only mildly elevated basal serum tryptase	Supports the presence of clonal mast cell disease and refines risk stratification beyond tryptase alone
Hereditary α-tryptasemia	Persistently elevated basal serum tryptase and/or severe reactions without overt mastocytosis	Should be considered as a risk-modifying trait, particularly in patients with severe reactions and unexplained tryptase elevation

These factors do not replace allergy diagnostics but refine risk stratification beyond sensitization testing alone. Importantly, a normal basal serum tryptase level does not exclude clinically relevant mast cell disease.
